# What is the adherence to an exercise intervention during (neo-)adjuvant chemotherapy among Swedish patients with breast cancer? Data from the Phys-Can randomised controlled trial

**DOI:** 10.1136/bmjopen-2025-105540

**Published:** 2026-05-07

**Authors:** Anna Henriksson, Andreas Stenling, Anne-Sophie Mazzoni, Katarina Sjövall, Sussanne Börjeson, Sveinung Berntsen, Christopher G Bean, Laurien M Buffart, Karin Nordin, Ingrid Demmelmaier

**Affiliations:** 1 Department of Women and Children’s Health, Uppsala University, Uppsala, Sweden; 2 Department of Public Health and Caring Sciences, Uppsala University, Uppsala, Sweden; 3 Department of Psychology, Umeå University, Umea, Sweden; 4 Department of Sport Science and Physical Education, University of Agder, Kristiansand, Norway; 5 Faculty of Health Sciences, Kristianstad University, Kristianstad, Sweden; 6 Department of Oncology and Department of Health, Medicine, and Caring Sciences, Linköping University, Linkoping, Sweden; 7 School of Psychology, Adelaide University, Adelaide, South Australia, Australia; 8 Department of Medical BioSciences, Radboud University Medical Center, Nijmegen, Netherlands

**Keywords:** Breast tumours, Exercise, CHEMOTHERAPY

## Abstract

**Objectives:**

While exercise adherence is known to vary during cancer treatment, little is known about what predicts these changes during chemotherapy or within individual treatment cycles for breast cancer. We examined changes in adherence and its predictors (1) across chemotherapy treatment and (2) within treatment cycles in women undergoing (neo-)adjuvant chemotherapy for breast cancer.

**Design:**

This study is based on data from the Phys-Can multicentre parallel randomised trial.

**Setting:**

The exercise intervention was conducted at public gyms in three Swedish university cities.

**Participants:**

178 women undergoing (neo-)adjuvant chemotherapy without any chemotherapy treatment delays and had any adherence data were included in the analysis.

**Interventions:**

Participants in the Phys-Can trial were randomised to either high or low-to-moderate intensity combined endurance and resistance training.

**Primary outcome:**

The primary outcome variable for this secondary analysis of Phys-Can trial data was adherence to endurance and resistance training. Bayesian multilevel growth curve models were used to examine adherence to resistance and endurance training throughout the chemotherapy treatment period and within chemotherapy cycles. Potential predictors of adherence included exercise intensity, chemotherapy dose, muscle strength, body mass index, cardiorespiratory fitness, fatigue and age. Results are reported with 95% credibility intervals (CrIs).

**Results:**

Adherence to endurance and resistance training declined on average across the chemotherapy treatment by 1% (95% CrI −1.5, −0.5) and 5.2% (95% CrI −6.8, −3.6), respectively, per week. Adherence decreased within the chemotherapy treatment cycle by 2.4% for endurance (95% CrI −4.2, −0.7) and 6.1% (95% CrI −8.2, −4.1) for resistance training, respectively. Higher baseline fitness predicted better adherence to endurance exercise (*β*=1.2, 95% CrI 0.1, 2.3), while high-intensity training predicted a steeper decline (*β*=−1.2, 95% CrI −2.2, 0.2). No significant predictors were found for adherence to resistance training over time.

**Conclusions:**

Women with breast cancer may require additional support to maintain exercise adherence during the later stages of chemotherapy and during the second and third weeks of their chemotherapy cycles. Those with lower pretreatment fitness levels may benefit from more intensive support to sustain engagement in exercise.

The Phys-Can trial was registered in Clinical trials: ClinicalTrials.gov NCT02473003,

**STRENGTHS AND LIMITATIONS OF THIS STUDY**

- This study is based on data from a high-quality multicentre randomized controlled trial and contains detailed adherence reporting.
- Exclusion of participants with treatment delays or non-standard chemotherapy regimens reduces generalisability.
- The relatively small study sample may have reduced statistical power to detect smaller effects on potential predictors of adherence.
- Because the interval between study inclusion and treatment start was short, data for the first chemotherapy cycle were limited.

## Introduction

 It is well established that exercise, defined as structured and planned physical activity aimed at maintaining or enhancing physical fitness or health,^1^ provides many benefits for patients undergoing oncological treatment. For instance, exercise can reduce side effects such as cancer-related fatigue, improve or maintain physical fitness and health-related quality of life, and has been associated with disease-free survival in patients with breast and colon cancer.^2^,^4^ Conversely, adhering to exercise recommendations is challenging for many patients during treatment.^5^

Adherence to exercise intervention protocols can be defined as the extent to which an individual follows a protocol with regard to the prescribed combined exercise frequency, intensity, time and type.^6^ Several factors influencing exercise adherence among patients with cancer have been identified in systematic reviews and meta-analyses, reflecting a complex interplay of biological, socioeconomic and medical factors.^7 8^ Sociodemographic, medical and physical factors also appear to play a significant role. High adherence has been associated with extensive prior exercise habits, higher cardiorespiratory fitness (measured as maximal oxygen uptake (VO_2_max)) and proximity to a rehabilitation centre.^7^ Additionally, sex differences have been documented; women, especially those with breast cancer, generally demonstrate lower adherence than men.^8^ In contrast, psychological factors appear to have less impact.^7^ However, research on exercise adherence among women with breast cancer undergoing chemotherapy is limited, highlighting the need for a better understanding of adherence patterns and influencing factors. Studies investigating attendance rates (ie, adherence to exercise frequency) to supervised resistance or endurance training during (neo-)adjuvant chemotherapy suggest that adherence is highest during the initial weeks of training. In one randomised controlled trial (n=122), attendance rates exceeded 60% for endurance training and 50% for resistance training during the first 6 weeks.^9^ However, adherence declined as chemotherapy progressed, with higher attendance during cycles 1–2 compared with cycles 3–8.^6^ In another study,^10^ a periodised exercise approach tailored to the cyclical nature of chemotherapy side effects was associated with improved adherence among 27 patients. However, no study has yet examined whether exercise adherence fluctuates within individual chemotherapy cycles.

Our research group previously found that higher age, higher cardiorespiratory fitness, higher quality of life and less physical fatigue at baseline among patients with breast, prostate and colorectal cancer undergoing (neo-)adjuvant treatment were positively associated with adherence to the Phys-Can randomised controlled trial, which includes 6 months of resistance training and endurance training.^11^ However, these findings describe overall adherence across the entire intervention and do not address how adherence may change during chemotherapy. It remains unknown how adherence fluctuates across the course of chemotherapy or within individual chemotherapy cycles, when symptoms and treatment burden may vary substantially. Moreover, it is not clear which patient and treatment-related factors might explain within-treatment changes in adherence. This knowledge will increase our understanding of when adherence is most challenging and which patients may need additional support. Therefore, the aims were to examine changes in exercise adherence and its predictors (1) across chemotherapy treatment and (2) within treatment cycles in women undergoing (neo-)adjuvant chemotherapy for breast cancer, participating in a 6 month exercise trial.

## Material and methods

This study uses data from the Swedish Phys-Can multicentre randomised controlled trial, which was conducted at three university cities: Linköping, Lund and Uppsala. Details of the trial procedures have been described previously.^12 13^ Briefly, the aim of the Phys-Can trial was to investigate the effects of exercise intensity and behaviour change support on cancer-related fatigue with a 2×2 factorial design, and these results have been published previously.^13^ Five hundred seventy-seven participants undergoing (neo-)adjuvant cancer treatment for breast, prostate or colorectal cancer were consecutively included and randomised to 6 months of combined endurance and resistance training at either high intensity (HI) or low-moderate intensity (LMI), with or without addition of behaviour change support. For this study of adherence during chemotherapy, we used the Phys-Can data from women with breast cancer, as it is one of the most prevalent cancers in women. In addition, because our research group previously found no effect of behaviour change support on exercise adherence, we compared the groups solely on training intensity.^14^

### Patient and public involvement

Patient representatives helped review patient information, inclusion routines and patient reported outcomes used in the Phys-Can trial prior to the start of the study.

### Participants

Participants included in the present analysis were women with breast cancer receiving (neo-)adjuvant (ie, treatment provided for reducing tumour burden prior to curative surgery) or adjuvant (ie, treatment provided after curative surgery for reducing risk of cancer relapse) chemotherapy. Exclusion criteria were as follows: inability to perform activities of daily living, disabilities that prevented physical activity, conditions that affected cognitive ability (ie, dementia and severe psychiatric illness), cardiopulmonary conditions preventing physical activity (ie, severe obstructive pulmonary disease and severe heart failure), breast cancer stage IIIb–IV, incomplete or recent cancer treatment (ie, treatment completed less than 12 months ago or not recovered from previous treatment), body mass index (BMI)<18.5, fibromyalgia and pregnancy. Patients deemed eligible by the treating oncologist or surgeon were subsequently provided with oral and written information regarding the Phys-Can trial during a planned visit to an oncology or surgery clinic before the start of treatment. Those who agreed to participate signed a consent form prior to baseline data collection.

### Exercise intervention

The exercise intervention was initiated at the start of the (neo-)adjuvant chemotherapy treatment and continued for 6 months. Thus, the present study includes exercise adherence data for the 4-month chemotherapy treatment period. Each supervised resistance training session lasted approximately 60–90 min, and all sessions were supervised by qualified and experienced coaches (physiotherapists or personal trainers). A 6-week familiarisation period for resistance training and 3 weeks for endurance training preceded the start of the exercise programme according to the protocol. The familiarisation period entailed a learning period where the participants got familiarised with the exercise protocol and gradually increased in the intensity and time until the target exercise volume was reached.^12^

The supervised resistance training was performed at public gyms free of charge. It consisted of two sessions per week and included three exercises for the upper extremities and three for the lower extremities. After the initial familiarisation period, the participants randomised to HI exercised with three sets of six or 10 repetitions at maximum muscle strength (repetition maximum (RM)) and continued to failure in the last set of each exercise. The participants randomised to LMI exercised at 50% of 6 RM or 10 RM, with three sets of 12 or 20 repetitions per set, respectively. Exercise intensity, that is, training load and progression, was based on repeated testing of 6 and 10 RM every 4–6 weeks in all exercises. Coaches recorded target weights in a logbook prior to each exercise session and provided feedback at each session.

The home-based endurance training at HI consisted of 2-min interval sessions with a gradually increasing number of intervals (typically increasing from 5 to 10) performed twice per week, while LMI endurance training consisted of 150 min of self-selected activity weekly, such as walking or biking. High-intensity intervals consisted of 2 min of exercise (ie, running, cycling and walking uphill) at 80%–90% of heart rate reserve (HRR), followed by 2 min of active rest. Progression began at five intervals, with added intervals over time until a maximum of 10 intervals was reached. Low to moderate intensity consisted of walking or cycling in bouts of at least 10 min at 40%–50% of HRR. HRR was determined for each participant at baseline with a VO_2_max test. Participants wore heart rate monitors and recorded their exercise in a logbook. Coaches reviewed the pulse files from the heart rate monitors and logbooks to monitor exercise intensity and overall adherence and provided feedback to the participants.

### Data collection

#### Baseline background variables

Baseline age was gathered from medical records. Self-reported data on education and co-morbidities (ie, any physical or psychological conditions that were medicated for) were gathered at baseline with a study-specific questionnaire. Comorbidities were categorised by number of present comorbidities.

#### Outcome variable

##### Adherence to prescribed exercise

Adherence to exercise was the primary outcome variable of this study and defined as performed percentage of prescribed exercise volume (ie, combined exercise frequency, time and intensity) and described for resistance and endurance training separately. Adherence data were based on logbook data for resistance training and a combination of logbook data and pulse file data for endurance training. Exercise intensity (ie, training load) and progression were based on repeated testing of 6 and 10 RM every 4–6 weeks in all exercises. Exercise volume was calculated as percentage of performed exercise volume/prescribed exercise volume by each week, that is, for resistance training as weight×repetitions, summarised across all exercises and sessions and for HI endurance training as number of intervals×interval duration, summarised across all sessions. Exercise volume for LMI endurance training was calculated as percentage of 150 min of low to moderate intensity per week. For resistance training and HI endurance training, the familiarisation period was not included since exercise volume could not be calculated. Phys-Can participants increased their planned exercise volume over the intervention period (progression). As a result, the required exercise volume to achieve 100% adherence also increased over time as the participants became stronger. If a participant had to temporarily lower their weights in relation to the latest training session weights or 6 and 10 RM test results due to side effects, this was considered a decrease in adherence.

### Predictor variables

#### Chemotherapy dose

Information regarding the chemotherapy dose as well as the start and end date of treatment was gathered from the participants’ medical records. Participants received standardised chemotherapy according to national clinical cancer care guidelines,^15^ most commonly a combination of fluorouracil 500–600 mg/m^2^ (F) and/or only epirubicin 75–100 mg/m^2^ (E) and cyclophosphamide 500–600 mg/m^2^ (C) sequenced with docetaxel 75–100 mg/m^2^ (D). Chemotherapy was given in six cycles, where each cycle was 21 days (ie, approximately 3 weeks). Chemotherapy dose was dichotomised into high (ie, F/C 600 mg/m^2^, E/D 100 mg/m^2^) and low (F/C 500 mg/m^2^, E/D 75–80 mg/m^2^) doses. Chemotherapy cycles that were postponed for more than 7 days were considered a treatment delay.

#### Baseline muscle strength

Muscle strength was assessed by a 1 RM test at baseline. The test was performed after the 6-week familiarisation period and was assessed in seated single-leg press (left and right leg tested separately) and seated chest press.^13^ Results were summed together and expressed as total strength in kilograms (kg).

#### Baseline body mass index

Body weight and height were measured at baseline, and BMI was calculated as weight in kg divided by height².

#### Baseline cardiorespiratory fitness

Cardiorespiratory fitness was measured as a maximal oxygen uptake (mL oxygen/kg) with a treadmill test until exhaustion (VO_2_max test) at baseline. A modified Balke protocol (ie, a graded treadmill test) was used, and details have been previously published.^16^ A test was considered valid if two of the following criteria were met: (1) testing staff judged it as a maximal test, (2) Borg perceived exertion^17^ at 17 or above and (3) respiratory exchange ratio was at or above 1.1.

#### Baseline physical fatigue

Physical fatigue was measured with Multidimensional Fatigue Inventory (range 4–20)^18^ at baseline. Physical fatigue was the main outcome of the Phys-Can trial.^12 13^

### Statistical analysis

A baseline comparison on background characteristics was performed using independent-samples t-tests for continuous variables, and categorical variables were compared using χ^2^ tests and Fisher’s exact test. A significance level of 0.05 was used.

Mplus V.8.11 (Muthén & Muthén, 1998–2017) was used to conduct the statistical analysis. First, we used Bayesian multilevel growth curve models to examine the level (ie, the intercept) and change over time (ie, the slope) in exercise adherence of resistance and endurance training across the entire 18-week treatment period (models shown in online supplemental file 1). The intercept (ie, level of adherence in percentage) was placed at week 3 for endurance training adherence and week 6 for resistance training adherence because many participants had not started to engage in regular training until these weeks, and therefore, these weeks provide a better estimate of early/initial levels of adherence compared with earlier in the treatment period. Linear and quadratic random slopes were examined, and model comparisons were conducted using the deviance information criterion (DIC). A model with lower DIC indicates a better model fit compared with a model with higher DIC. Furthermore, we only retained a quadratic slope if the 95% credibility interval (CrI) for the quadratic slope mean did not include 0. Time was represented by weekly assessments; hence, the linear slope indicates the average rate of change per week, and the quadratic slope represents the rate of acceleration (ie, indicates if rate of change speeds up or slows down). Both average levels and variances (σ²) are reported for the intercept and slope factors. Average levels are fixed effects representing the mean when pooling all individuals within the sample, and the variances are random effects representing interindividual differences around the average levels.

Second, exercise intensity, chemotherapy dose, baseline muscle strength, baseline BMI, baseline cardiorespiratory fitness, baseline physical fatigue and age were added as between-person level predictors of the intercept (ie, level of adherence) and slope factors (ie, rate of change in adherence).

Third, multilevel growth models were used to examine changes in endurance and resistance training adherence within the 3-week treatment cycles, while accounting for the overall change across the entire 18-week treatment period (ie, detrending).^19 20^ Data from all chemotherapy cycles were collapsed and calculated for chemotherapy cycle week 1, cycle week 2 and cycle week 3, respectively. Only linear random slope models were estimated; hence, the slope represents the change per treatment cycle week averaged across all six treatment cycles. The intercept (ie, level of adherence) was placed at the first treatment cycle week and represents the level of training adherence in the first treatment cycle week averaged across all six treatment cycles.

Fourth, exercise intensity, chemotherapy dose, baseline muscle strength, baseline BMI, baseline cardiorespiratory fitness, baseline physical fatigue and age were added as between-person level predictors of the intercept (ie, level of adherence in the first treatment cycle week) and slope (ie, rate of change in adherence within treatment cycle weeks) factors.

All parameter estimates are presented with 95% CrI. If the 95% CrI of the parameter estimate did not include 0, it is interpreted as credible and statistically significant.^21^ For further description of the statistical analysis, see the supplementary material.

### Results

In the Phys-Can trial, 276 women with breast cancer planned for neo-adjuvant or adjuvant chemotherapy were included.^13^ Of these, a total of 178 participants (table 1) receiving chemotherapy in six 3-week cycles without treatment delays and having exercise adherence data for endurance training (n=174) and/or resistance training (n=171) were included in the statistical analysis. 12.9% of participants received neo-adjuvant chemotherapy (n=23). A baseline comparison of exercise intensity groups showed no statistically significant differences in medical variables; however, the low-intensity group had a higher proportion of participants with a university education (table 1).

**Table 1:** Baseline characteristics

	High-intensity exercise (n=82)	Low-moderate intensity exercise (n=96)	Total (n=178)	P value
Age				
Mean (SD)	53.4 (10.3)	52.2 (10.3)	52.8 (10.3)	0.43
Education				0.05
Compulsory school	5 (6.1%)	6 (6.3%)	11 (6.2%)	
Upper secondary school	30 (36.6%)	19 (19.8%)	49 (27.5%)	
University	43 (52.4%)	66 (68.8%)	109 (61.2%)	
Missing data	3 (3.7%)	1 (1.0%)	4 (2.2%)	
Comorbidities				0.10
None	32 (39.0%)	43 (44.8%)	75 (42.1%)	
One comorbidity	21 (25.6%)	24 (25.0%)	45 (25.3%)	
Two comorbidities	16 (19.5%)	17 (17.7%)	33 (18.5%)	
Three or more comorbidities	4 (4.9%)	5 (5.2%)	9 (5.1%)	
Missing data	9 (11.0%)	7 (7.3%)	16 (9.0%)	
BMI (kg/m^2^)				0.39
Mean (SD)	25.4 (4.4)	24.8 (4.0)	25.1 (4.2)	
Missing data	4 (4.9%)	5 (5.2%)	9 (5.1%)	
VO_2_max (mL/min/kg)				0.10
Mean (SD)	31.3 (7.0)	31.3 (7.0)	31.3 (7.0)	
Missing data	4 (4.9%)	4 (4.2%)	8 (4.5%)	
Total 1 RM strength (kg)				
Mean (SD)	41.8 (13.0)	41.0 (14.2)	41.3 (13.6)	0.70
Missing data	9 (5.1%)	12 (6.7%)	21 (11.8%)	
Chemotherapy treatment				
Adjuvant	70 (85.4%)	85 (88.5%)	155 (87.1%)	0.69
Neo-adjuvant	12 (14.6%)	11 (11.5%)	23 (12.9%)	
FEC high dose	43 (52.4%)	62 (63.5%)	104 (58.5%)	
FEC low dose	32 (39.0%)	34 (35.4%)	66 (37.1%)	
Docetaxel high dose	38 (46.3%)	40 (41.7%)	78 (43.8%)	
Docetaxel low dose	37 (45.1%)	55 (57.3%)	92 (51.7%)	
Capecitabine	7 (8.5%)	1 (1.0%)	8 (4.5%)	
MFI physical fatigue				
Mean (SD)	11.0 (4.2)	10.8 (4.0)	10.9 (4.1)	0.78

BMI, body mass index; FEC, fluorouracil 500–600 mg/m2 and/or only epirubicin 75–100 mg/m2 – cyclophosphamide 500–600 mg/m2; MFI, Multidimensional Fatigue Inventory (range 4–20); 1 RM, 1 repetition maximum; VO_2_max, cardiorespiratory fitness measured with maximum oxygen uptake.

#### Exercise adherence across the chemotherapy treatment period and its predictors

**Figure 1:**
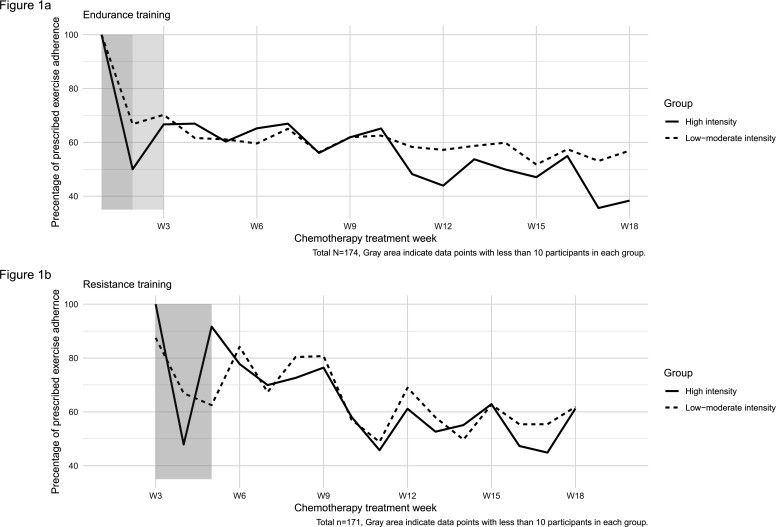
Exercise adherence during chemotherapy treatment. Points represent the observed mean adherence (%) at each week across the 18-week study period, based on participants with available data at that time point.

The observed mean adherence across the entire 18-week treatment period is shown in figure 1, and tables 1 and 2 in the Online supplemental file 1 show results from multilevel growth models describing these changes. A linear change model best represented the data for endurance training, whereas a quadratic change model best represented the data for resistance training.

**Table 2:** : Predictors of change in endurance training adherence across the treatment period

	Level of adherence week 3 (intercept)	Change over time (linear slope)
	*β (LL, UL*)	*β (LL, UL*)
Exercise intensity (high)	4.56 (−6.24, 15.47)	**−1.16 (−2.15, −0.21**)
Chemotherapy dose (high)	−6.25 (−19.20, 6.48)	0.15 (−1.03, 1.33)
Muscle strength	0.09 (−0.46, 0.65)	−0.00 (−0.05, 0.05)
BMI	−0.60 (−2.43, 1.24)	−0.10 (−0.26, 0.07)
Cardiorespiratory fitness	**1.19 (0.10, 2.28**)	−0.03 (−0.13, 0.07)
Fatigue	0.38 (−1.06, 1.81)	−0.05 (−0.18, 0.08)
Age	0.45 (−0.25, 1.16)	−0.03 (−0.10, 0.03)

Bold estimates indicate statistically significant effects. Average level of adherence at chemotherapy week 3 is represented by the random intercept and the change over time by the random linear slope.

BMI, body mass index; LL, lower level 95% credible interval; UL, upper level 95% credible interval.

For endurance training (see tabel 1, model 2 in the online supplemental file 1), the average level of adherence at week 3 was 64% (95% CrI 58.71, 69.30, σ^2^=824.67, 95% CrI 585.15, 1139.54). Endurance training adherence decreased 1% per week (linear slope=−0.99, 95% CrI −1.47, −0.51, σ^2^=5.60, 95% CrI 3.69, 8.15). As seen in table 2, higher fitness level at baseline positively predicted higher levels of endurance training adherence at week 3 (*β*=1.19%, 95% CrI 0.10, 2.28), whereas being in the high exercise intensity group predicted a steeper decline across the treatment period (*β*=−1.16%, 95% CrI −2.15, 0.21).

For resistance training (see table 2, model 3 in the online supplemental file 1), the average level of adherence at week 6 was 73% (95% CrI 68.79, 76.44, σ^2^=196.71, 95% CrI 109.64, 323.51). The average adherence decreased 5% per week (linear slope=−5.20, 95% CrI −6.85, −3.59, σ^2^=33.85, 95% CrI 17.38, 59.25) but levelled off across the treatment period (quadratic slope=0.38, 95% CrI 0.22, 0.54, σ^2^=0.30, 95% CrI 0.13, 0.57). None of the baseline predictors had statistically significant effects on levels of adherence at week 6 or changes in adherence of resistance training across the treatment period (table 3).

**Table 3:** Predictors of change in resistance training adherence across the treatment period

	Level of adherence week 6 (intercept) *β (LL, UL*)	Change over time (linear slope) *β (LL, UL*)	Acceleration/deceleration (quadratic slope) *β (LL, UL*)
Exercise intensity (high)	−1.78 (−9.72, 6.04)	−0.55 (−3.80, 2.72)	0.04 (−0.29, 0.37)
Chemotherapy dose (high)	−2.55 (−12.14, 7.15)	−0.91 (−4.87, 3.19)	0.00 (−0.40, 0.40)
Muscle strength	−0.08 (−0.51, 0.35)	0.04 (−0.14, 0.22)	−0.01 (−0.02, 0.01)
BMI	0.66 (−0.76, 2.05)	−0.05 (−0.62, 0.58)	0.00 (−0.06, 0.06)
Cardiorespiratory fitness	0.44 (−0.43, 1.30)	0.33 (−0.03, 0.69)	−0.02 (−0.06, 0.01)
Fatigue	0.38 (−0.63, 1.35)	0.01 (−0.41, 0.43)	−0.01 (−0.05, 0.03)
Age	−0.05 (−0.58, 0.47)	0.06 (−0.15, 0.28)	−0.01 (−0.03, 0.01)

Average level of adherence at chemotherapy week 6 is represented by the random intercept and change over time by the random linear slope. Acceleration (ie, if rate of change speeds up or slows down) is represented by the random quadratic slope.

BMI, body mass index; LL, lower level 95% credible interval; UL, upper level 95% credible interval.

#### Exercise adherence within the chemotherapy cycles and its predictors

**Figure 2:**
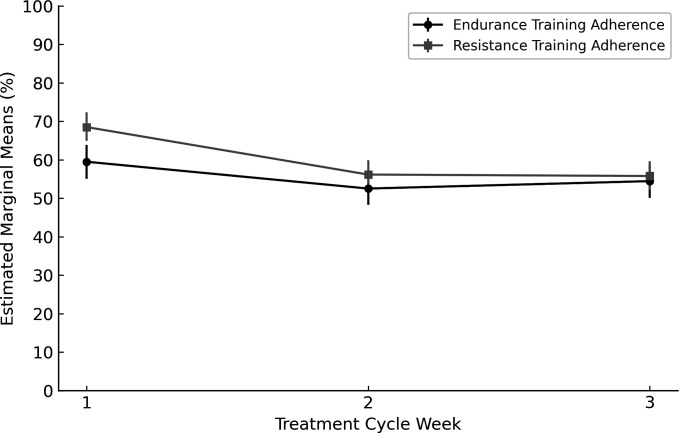
Model-estimated marginal mean adherence (%) for endurance and resistance training at weeks 1–3 within the chemotherapy treatment cycle. Error bars represent credible intervals around the model-estimated means.

Changes within the 3-week treatment cycles for endurance and resistance training adherence and predictors of training adherence within the 3-week treatment cycles are presented in table 4. Estimated marginal means within the treatment cycles show that most of the decrease occurred from the first to second week in both endurance and resistance training adherence (figure 2).

**Table 4:** Predictors of initial levels and changes in endurance and resistance training adherence within the 3-week treatment cycles

	Endurance training		Resistance training	
	Level of adherence Cycle week 1 (intercept)	Change over time (linear slope)	Level of adherence Cycle week 1 (intercept)	Change over time (linear slope)
	*β (LL, UL*)	*β (LL, UL*)	*β (LL, UL*)	*β (LL, UL*)
Exercise intensity (high)	−2.47 (−10.83, 5.93)	−3.06 (−6.32, 0.13)	−2.81 (−9.67, 4.00)	−0.12 (−4.33, 4.04)
Chemotherapy dose (high)	−6.69 (−16.89, 3.46)	1.84 (−2.41, 5.69)	−3.56 (−11.73, 4.62)	−3.15 (−8.17, 1.87)
Muscle strength	−0.03 (−0.47, 0.42)	0.10 (−0.07, 0.28)	−0.11 (−0.47, 0.25)	0.04 (−0.17, 0.25)
BMI	−1.40 (−2.84, 0.04)	−0.08 (−0.64, 0.46)	0.00 (−1.15, 1.16)	0.57 (−0.13, 1.28)
Cardiorespiratory fitness	0.84 (−0.03, 1.71)	0.06 (−0.28, 0.41)	**0.90 (0.21, 1.59**)	0.35 (−0.08, 0.77)
Fatigue	−0.41 (−1.50, 0.70)	0.34 (−0.12, 0.76)	−0.02 (−0.90, 0.84)	0.11 (−0.42, 0.65)
Age	0.10 (−0.43, 0.65)	0.07 (−0.15, 0.29)	−0.02 (−0.46, 0.41)	−0.03 (−0.30,0.24)

Bold estimates indicate statistically significant effects.

BMI, body mass index; LL, lower level 95% credible interval; UL, upper level 95% credible interval.

For endurance training adherence, the results showed an average linear decrease within the treatment cycle of 2% (95% CrI −4.16, −0.71, σ^2^=5.20, 95% CrI 0.52, 21.33). None of the baseline variables were statistically significant predictors of change in adherence within the 3-week treatment cycles (all the 95% CrIs included 0) for endurance training.

For resistance training, the average linear decrease in training adherence within the treatment cycles was 6% (95% CrI −8.21, −4.09, σ^2^=36.78, 95% CrI 8.70, 81.57). For resistance training, baseline fitness had a statistically significant and positive effect on the average level of training adherence in the first treatment cycle week (*β*=0.90, 95% CrI 0.21, 1.59).

### Discussion and conclusion

In the present study, we found that exercise adherence varied throughout the chemotherapy treatment and decreased over time for both resistance and endurance training. Furthermore, our results show that exercise adherence varies within chemotherapy treatment cycles, with a decline in the first week after chemotherapy that is maintained in the second and third weeks. For endurance training, higher cardiorespiratory fitness at baseline was associated with higher adherence at week 3, and being in the high-intensity group was associated with a steeper decline in endurance training adherence throughout the chemotherapy treatment, indicating that LMI may be more feasible to maintain during chemotherapy. The association between higher baseline cardiorespiratory fitness and higher adherence, as previously reported,^7 11^ suggests that individuals with lower cardiorespiratory fitness at the start of chemotherapy may need more support than fitter individuals. It also raises the question of whether patients with breast cancer planned for chemotherapy treatment may benefit from pre-treatment (ie, prehabilitation) and peri-operative (before and after surgery) exercise interventions to improve their cardiorespiratory fitness and support their ability to exercise during chemotherapy. A systematic review of pre-rehabilitation for women with breast cancer found an increase in 6-min walk test prior to surgery, suggesting improved aerobic fitness. However, it remains uncertain whether these improvements lead to better adherence to exercise during chemotherapy after surgery (ie, adjuvant treatment) because of the lack of long-term follow-up.^22^

For resistance training, none of our included potential predictors were associated with exercise adherence across the entire chemotherapy treatment period. Similarly, none of the potential predictors for adherence to endurance or resistance training were associated with the decline within chemotherapy cycles. However, we observed that higher baseline cardiorespiratory fitness was associated with higher resistance training adherence during the first week (week 1) of the chemotherapy cycle.

Patients receiving high-dose chemotherapy also often receive supportive therapies such as colony-stimulating growth factors, which reduce the severity and duration of neutropenia.^23^ This may explain why individuals receiving high-dose chemotherapy have similar levels of adherence to exercise than individuals receiving low-dose chemotherapy and should be explored in future research.

The results support the suggestion of Kirkham *et al*^10^ that adherence varies within cycles and align with previous research showing a decline over the treatment trajectory.^10 11 24^ Side effects from treatment may accumulate over time within each chemotherapy cycle, which may explain why it is more difficult to maintain adherence to high-intensity exercise throughout the chemotherapy treatment,^6^ especially unsupervised exercise such as our endurance training. We did not find an association between baseline physical fatigue and adherence. Increasing fatigue levels within chemotherapy cycles, or fatigue accumulated over the treatment period, may play a more important role in adherence than fatigue at the start of treatment^10^ and should be further investigated.

Our results indicate that during a week with an increased burden of treatment-related side effects, the exercise prescription could be changed by frequency, intensity and/or duration so that the exercise volume is maintained. Non-linear exercise prescription where exercise sessions are varied regarding intensity and duration (ie, includes both progression and recovery training sessions) has previously been suggested by Sasso *et al*.^25^ However, exercise interventions examining the effects of non-linear versus linear exercise prescription (ie, only including progression of training load) during chemotherapy for women with breast cancer are lacking. Furthermore, support for adherence, that is, behaviour change strategies such as action planning as well as intensified follow-ups during more challenging weeks may be useful. Exercise may offer patients short-term improvements of energy levels and reductions in nausea.^26 27^ Consequently, healthcare providers should inform and encourage patients to engage in physical activity, even when side effects are present. The support may also address instances when exercise is contra-indicated (eg, infections) or when exercise tolerance is very low (eg, extreme fatigue). This could include low-intensity physical activities instead of focusing on exercise; however, such strategies need to be evaluated in randomised trials.

### Strengths and limitations

To our knowledge, this is the first study to explore exercise adherence among women with breast cancer across the entire chemotherapy treatment period and within the chemotherapy cycles. The main strength of this study is the detailed reporting of adherence (ie, exercise volume for each week) during chemotherapy, based on data from a well-controlled randomised trial.^13^ The lack of observed effects on adherence in relation to chemotherapy dose, fatigue and age may be explained by the relatively homogenous sample included in the analysis (eg, only individuals with breast cancer who completed chemotherapy without treatment delays). Women with breast cancer receiving chemotherapy are generally younger than patients with prostate or colorectal cancer. Furthermore, the reduced number of individuals included in the analysis may have limited the power to detect some effects, particularly small ones.

For the present analysis, participants with treatment delays or chemotherapy regimens other than 3-week cycles were excluded. As a result, the generalisability of the findings is limited to patients who completed chemotherapy without delays. Individuals with treatment delays are likely to experience more severe treatment-related side effects and therefore lower adherence. For the same reason, the findings may not apply to patients receiving other types of treatments or treatment for other cancer diagnoses with more or less severe treatment-related side effects. Although gym memberships were provided, travel distances for participants living farther from the training sites could have affected adherence.^7^ Also, the low-intensity group also had a greater proportion of participants with a university education. These factors should be considered when interpreting the generalisability of our findings. Additionally, due to the short time between inclusion and start of treatment and inclusion of an exercise familiarisation period, many individuals began their chemotherapy before starting the exercise programme. As such, we had limited exercise volume data for the first weeks after chemotherapy initiation. We also had fewer data points for resistance training due to the longer familiarisation period (6 weeks for resistance training vs 3 weeks for endurance training).

On average, participants did not complete the full prescribed exercise dose. While this provides important insight into the feasibility of the intervention, it should not be interpreted as evidence that lower exercise volumes are sufficient for improving or maintaining outcomes. Evidence from a systematic review and meta-analysis of oncology exercise studies suggests a non-linear dose-response relationship for improving health-related quality of life, with the optimal exercise dose identified as 850 metabolic equivalents per minute per week.^28^ Yet, the optimal and minimal effective doses during cancer treatment are unclear. Future research should use well-powered randomised controlled trials to determine the lowest clinically effective exercise dose during chemotherapy on key outcomes such as cancer-related fatigue or cardiorespiratory fitness.

### Interpretation

Women with breast cancer may require additional support, such as intensified follow-up and/or individualised adjustment to the exercise programme to maintain exercise adherence as their treatment progresses, particularly during the second and third weeks of chemotherapy cycles. Furthermore, LMI endurance training may be more feasible to maintain throughout the chemotherapy treatment, and individuals with lower fitness prior to starting treatment may benefit from more intensive support, such as supervised exercise and behaviour change strategies. While this study provides valuable insights into exercise adherence during chemotherapy, further research is needed to evaluate non-linear exercise prescriptions and to examine adherence in broader cancer populations, including those with treatment delays.

## Supplementary material

10.1136/bmjopen-2025-105540 online supplemental file 1

## Data Availability

The datasets for the Phys-Can trial are not publicly available since the participants did not provide consent to share the data openly; however, de-identified datasets used are available from the corresponding author upon reasonable request.
