# Supplementary material for: What is the adherence to an exercise intervention during (neo-)adjuvant chemotherapy among Swedish patients with breast cancer? Data from the Phys-Can randomised controlled trial
Source: BMJ Open. 2026 May 7;16(5):e105540. doi: 10.1136/bmjopen-2025-105540 (PMC13157739; doi:10.1136/bmjopen-2025-105540)
Supplement: online supplemental file 1 [file bmjopen-16-5-s001.docx]

**SUPPLEMENTAL MATERIAL**

The Bayesian multilevel models were estimated using four Markov chain Monte Carlo (MCMC) chains and 20000 to 50000 iterations. The first half of the iterations are discarded as burn-in and remaining half is used to estimate posterior distributions of the parameters. We used the potential scale reduction factor (PSFR;(1)) to assess chain convergence and a low (e.g., < 1.05) and stable PSFR was used as evidence of chain convergence. All between-person predictors were grand-mean centered (2). The default noninformative prior specification in Mplus was used in all analyses (see Muthén & Muthén, 1998-2017 (3), for a description of the default noninformative prior specification in Mplus). We included all available data in the analyses, similar to the full information maximum likelihood (FIML) estimation. The Gibbs sampler treats the missing observations as unknown values to be estimated; the estimates will therefore be adjusted for missingness (4).

1. Brooks SP, Gelman A. General Methods for Monitoring Convergence of Iterative Simulations. J Comput Graph Stat. 1998 Dec;7(4):434–55.

2. Yaremych HE, Preacher KJ, Hedeker D. Centering categorical predictors in multilevel models: Best practices and interpretation. Psychol Methods. 2023 Jun;28(3):613–30.

3. Muthén B, Muthén L. Mplus. In: Handbook of item response theory. Chapman and Hall/CRC; 1998-2017. p. 507–18.

4. Asparouhov T, Muthén B. Weighted least squares estimation with missing data. Mplus Tech Append. 2010;2010(1–10):5.

Table S1

*Parameter Estimates from the Multilevel Growth Model of Adherence to Endurance Training Across the Chemotherapy Treatment Period*

| Endurance training | | | |
| --- | --- | --- | --- |
|  | Model 1 | **Model 2** | Model 3 |
|  | Random intercept | **Random linear slope** | Random quadratic slope |
| **Model for the**  **Means** |  |  |  |
| Level of adherence week 3 (Intercept) | 55.39  [51.38, 59.32] | 64.02  [58.71, 69.31] | 59.67  [51.94, 67.05] |
| Change over time (linear slope) |  | -0.99  [-1.47, -0.51] | 0.356  [-1.38, 2.13] |
| Acceleration (quadratic slope) |  |  | -0.08  [-0.18, 0.02] |
| **Model for the**  **Variance** |  |  |  |
| Intercept  Variance | 625.80  [493.69, 802.24] | 824.67  [585.15, 1139.54] | 966.28  [542.14, 1618.67] |
| Linear Slope  Variance |  | 5.60  [3.69, 8.15] | 39.91  [16.99, 74.93] |
| Quadratic Slope  Variance |  |  | 0.11  [0.04, 0.21] |
| Intercept-Linear  Covariance |  | -35.24  [-59.32, 16.60] | -108.03  [-244.97, -20.20] |
| Intercept-Quadratic  Covariance |  |  | 3.73  [-0.74, 10.62] |
| Linear-Quadratic  Covariance |  |  | -1.91  [-3.76, -0.66] |
| Residual  Variance | 1027.51  [967.67, 1090.76] | 921.55  [865.71, 981.73] | 892.93  [836.76, 953.92] |
| **DIC** | 22620.88 | 22463.95 | 22434.02 |
| *Note*. Bold estimates indicate best fit model. 95% Credible intervals within square brackets. DIC: deviance information criterion. | | | |

Table S2

*Parameter Estimates from the Multilevel Growth Model of Adherence to Resistance Training Across the Chemotherapy Treatment Period*

| Resistance Training | | | |
| --- | --- | --- | --- |
|  | Model 1 | Model 2 | **Model 3** |
|  | Random intercept | Random linear slope | **Random quadratic slope** |
| **Model for the**  **Means** |  |  |  |
| Level of adherence week 6 (Intercept) | 60.31  [57.12, 63.44] | 68.49  [65.07, 71.92] | 72.52  [68.79, 76.44] |
| Change over time (linear slope) |  | -1.66  [-2.24, -1.11] | -5.20  [-6.85, -3.59] |
| Acceleration (quadratic slope) |  |  | 0.38  [0.22, 0.54] |
| **Model for the**  **Variance** |  |  |  |
| Intercept  Variance | 320.13  [237.92, 430.02] | 191.83  [107.93, 308.32] | 196.71  [109.64, 323.51] |
| Linear Slope  Variance |  | 4.95 [2.52, 8.31] | 33.85  [17.38, 59.25] |
| Quadratic Slope  Variance |  |  | 0.30  [0.13, 0.57] |
| Intercept-Linear  Covariance |  | 2.74  [-12.29, 13.95] | -15.09  [-55.63, 14.28] |
| Intercept-Quadratic  Covariance |  |  | 1.88  [-1.18, 5.82] |
| Linear-Quadratic  Covariance |  |  | -2.95  [-5.48, 1.32] |
| Residual  Variance | 1168.23  [1092.19, 1249.84] | 1074.69  [1002.25, 1154.96] | 1020.95  [948.89, 1098.94] |
| **DIC** | 18154.64 | 18049.45 | 17999.52 |
| *Note*. Bold estimates indicate best fit model. 95% Credible intervals within square brackets. DIC: deviance information criterion. | | | |

´
