# Supplementary material for: What is the adherence to an exercise intervention during (neo-)adjuvant chemotherapy among Swedish patients with breast cancer? Data from the Phys-Can randomised controlled trial
Source: BMJ Open. 2026 May 7;16(5):e105540. doi: 10.1136/bmjopen-2025-105540 (PMC13157739; doi:10.1136/bmjopen-2025-105540)
Supplement: Supplementary file 2 [file bmjopen-2025-105540.reviewer_comments.pdf]

PEER REVIEW HISTORY

BMJ Open publishes all reviews undertaken for accepted manuscripts. Reviewers are asked to complete a checklist review form and are provided with free text boxes to elaborate on their assessment. These free text comments are reproduced below.

ARTICLE DETAILS

Title (Provisional)

What is the adherence to an exercise intervention during (neo-)adjuvant chemotherapy among Swedish patients with breast cancer? - data from the Phys-Can Randomised Controlled Trial

Authors

Henriksson, Anna; Stenling, Andreas; Mazzoni, Anne-Sophie; Sjövall, Katarina; Börjeson , Sussanne; Berntsen, Sveinung; Bean, Christopher G; Buffart, LM; Nordin, Karin; Demmelmaier, Ingrid

---

VERSION 1 - REVIEW

Reviewer 1

Name Okuyama, Ayako

Affiliation St Luke's International University, Center for Cancer Control and Information Services

Date 31-Jul-2025

COI None

Adherence to an exercise intervention during (neo-) adjuvant chemotherapy in patients with breast cancer-data from the Phys-Can RCT

Thank you for giving me an opportunity to review the manuscript. This study deals with the interesting topic of exercise in patients undergoing chemotherapy. However, there are concerns about publishing the results of the RCT.

Major concern

1) By way of background, the authors note that research on exercise adherence in breast cancer patients is limited. However, the GAPs, such as what is known and what would be useful to investigate, are not adequately communicated to the reader.

2) It is not clear what is being evaluated as the primary outcome in this study. It says it wants to look at the effects of fatigue, or describes it as exploring factors, which confuses the reader.

3) In the methods, it is necessary to state what was assessed as the primary outcome and how the sample size was estimated.

4) Although the exclusion criteria for selecting subjects have been described, it is necessary to first describe the selection criteria in the first place. Breast cancer may be treated with a variety of chemotherapies. The purpose of chemotherapy (e.g., prevention of recurrence, arrest of progression, etc.) may also vary. It is necessary to clearly state which patients were selected for intervention. Furthermore, older patients may have difficulty exercising in the first place. It also needs to be clearly stated how the patients were selected, taking age and other factors into consideration.

5) It is also necessary to explain who guided the intervention and how it was performed by the patient.

6) Please explain the method of recruiting the target patients.

7) Please clarify this objective and then consider the discussion.

Minor

Why not add a graph to show sample assignments and dropouts?

**Reviewer**	**2**
**Name**	**Dylke, Elizabeth S**
**Affiliation**	**The University of Sydney**
**Date**	**18-Sep-2025**
**COI**	**None**

There is growing evidence of the benefits of undertaking exercise during treatment for breast cancer and understanding the barriers to completing physical activity are worthy of consideration. The treatment for cancer is physically difficult, potentially leading to challenges in maintaining physical activity throughout chemotherapy treatment.

This manuscript provides some novel information to the field but would benefit from some clarifications to provide better understanding for the reader about the methods and patients as well as something other things to consider in the discussion.

General feedback

Through the article the phrasing used is “(neo-)adjuvant” likely reflecting that some patients had their chemotherapy before surgery and others had it after surgery. However, its actually never explained why that term is used, nor is it described in the participant table how many people had neoadjuvant chemotherapy and how had many adjuvant chemotherapy. This should be clarified and explained. For example, if some participants are starting

chemotherapy not long after surgery, this may impact on their baseline fitness and what they are able to do.

Specific feedback

Methods

The manuscript needs a statement about where the ethics for this project was attained from

Exercise intervention

I found this section a bit difficult to understand, without visiting the other paper on the study. I think the part that needed a bit more explanation is the impact of the familiarisation program as it impacts on the results later.

Pg 6, line 137-139: Also, a six-week familiarisation period for resistance training and three-weeks for endurance training, preceded the start of the exercise program according to the protocol 11.

- Please confirm that taking part in this familiarisation program would at times mean that the exercise intervention didn't start until after the patient had undergone a round or maybe 2 of chemotherapy. What happened during the familiarisation sessions? What was the adherence through this period?

Pg 6: Supervised resistance training- can you confirm how long the participants were supervised for?

Pg 7: High-intensity intervals at 80-90% HRR is very high for many healthy participants, let alone those going through a major treatment like chemotherapy. What was the justification for this level of intensity? I also think that in the discussion the impact of the high level of intensity on adherence may be worth considering. (I say this with not only experience as an exercise professional but also as a runner who has gone through chemotherapy for breast cancer and was able to maintain running through it).

Line 176-178: If a participant had to temporarily lower their weights due to side effects, this was considered a decrease in adherence.

- I think more explanation for this decision is needed; if they were able to meet the volume/intensity prescribed based on their current abilities (likely decreased to ensure they were safe in doing the exercise) why is this considered a decrease in adherence. Particularly in a population where they are, because of the treatment they are undertaking, being systematically weakened, it feels like only considering progression as adherence is unreflective of realistic goals for patients. I'd be curious about what percentage of "non-adherent" patients this applied to in your sample and if you considered people doing the dosage you prescribed them, even if it did have to be decreased due to treatment side effects, what the difference in your findings would be.

Results

The results are a bit difficult to follow and seem to have varying numbers that are hard to rectify across different sections.

For example:

Line 251-253: the mean adherence for resistance training across chemo was 58% (39) for HI and 62 % (38) for LMI

Line 268- 272: For resistance training (see Table S2, Model 3) the average level of adherence at week 6 was 73% (, 95% CI [68.79, 76.44], ⍰2 = 196.71, 95% CI [109.64, 323.51]). The average adherence decreased 5 % per week (linear slope = -5.20, 95% CI [-6.85, -3.59], ⍰2 = 33.85, 95% CI [17.38, 59.25]) but levelled off across the treatment period (quadratic slope = 0.38, 95% CI [0.22, 0.54], ⍰2 = 0.30, 95% CI [0.13, 0.57]).

- I understand that because of the familiarisation period, week 6 is the starting point for a lot (but I'm not sure how many) of participants (does this correspond to the end of the 2 round of chemo- I don't really understand how the weeks compare across individuals in the study). Starting at 73% adherence and then decreasing at 5% per week across 12 weeks, seems like it should end at a lower overall average adherence of 58%/62%. What do you mean that the decrease in adherence levelled off across the treatment period in reference to the average decrease?

Why was baseline fatigue the factor that was included in the determination of factors that may impact adherence? Was there differences in baseline fatigue between those who had adjuvant v neo-adjuvant chemo? Were there differences in adherence between those who had adjuvant v neo-adjuvant chemo?

Discussion

General comments- I think your adherence rates are pretty impressive. It might be good to also mention what adherence rates for this type of program (or physical activity programs) is for healthy people of a similar age. I think this discussion should actually be scaffolded in more positive sense. More details on how much supervision/support participants got during the trial would help others know how implementable this approach would be.

Also as mentioned earlier, commenting on the impact of the type of program (particularly for the HI endurance program, which appeared to have a greater decrease in adherence across the time period) would be worthwhile. This is a very tough program that you've asked participants to do.

Line 319-321: Increasing fatigue levels within chemotherapy cycles, or fatigue accumulated over the treatment period, may play a more important role in adherence than fatigue at the start of treatment

- As this study was designed to investigate fatigue, can you look into this with your data?

Other limitations- other chemotherapies are also given in 3 weekly cycles but may be associated with other/more barriers to maintaining exercise levels due to side effects associated with them

---

**Reviewer**	**3**
**Name**	**Gresham, Gillian**
**Affiliation**	**Cedars-Sinai Medical Center Samuel Oschin Comprehensive Cancer Institute, Cancer Research Center for Health Equity**
**Date**	**22-Sep-2025**
**COI**	**None**

Thank you for the opportunity to review this manuscript. The topic is interesting and relevant. However, major revisions are needed to improve the clarity of the manuscript, the quality of the writing, and the overall reporting of methods and results. My detailed comments are outlined below:

Title

• The title could be refined to better reflect the study aims and findings.

Abstract

• Methods: Consider presenting the study design, data source, and study population first, followed by details of the statistical analysis. More information should also be provided about the intervention, control group, and outcomes assessed.

• Results: Please clarify whether results reflect differences between study arms or are reported across the entire sample.

Strengths and Limitations

• The current statement, "Novel and detailed adherence reporting providing detailed insights," is vague. Please clarify what novel insights are provided and how they add to existing literature.

Manuscript

Introduction

• The writing throughout could be improved to enhance clarity and flow, and typographical errors should be corrected.

• The aims should be framed within the broader context of the Phys-Can multicenter trial, which should be introduced earlier in the introduction with appropriate references.

Methods

• It is unclear whether this is a secondary analysis of the Phys-Can trial or a prospective sub-study built into the trial. Please clarify this distinction, including whether additional consent was obtained for this subset of participants.

• Eligibility criteria should be clarified: were they the same as the parent trial or different?

• Intervention details need further elaboration:

o The manuscript states the intervention was supervised at a gym, but by whom?

o How was adherence monitored and tracked?

o How were individual exercise intensity levels assessed and adjusted?

• Outcome variables: Please clarify how adherence was assessed (e.g., self-report, physiologist evaluation) and how it was analyzed. Were any objective measures of physical activity or adherence assessed?

• Predictor variables: Consider whether chemotherapy timing within cycles was evaluated. Performance status (e.g., KPS or ECOG), treatment side effects/toxicity, and baseline PROs or quality of life measures could also be important predictors and should be addressed.

Results

• Results should be described in greater detail overall.

• A CONSORT-style flow diagram would strengthen the manuscript by showing the number of participants from the Phys-Can trial, those included in the present analysis, and reasons for exclusion (especially if different from the parent trial)

• Baseline characteristics should be summarized in the text (Table 1) and compared across exercise groups.

• More detail is needed regarding baseline predictors (e.g., whether baseline fatigue was associated with outcomes).

• Were longitudinal changes in predictors such as fatigue or muscle strength assessed across chemotherapy cycles, and were these associated with adherence outcomes?

• Sentence at lines 331–332 is unclear and incomplete and should be revised.

Discussion/Conclusion

• The discussion would benefit from deeper comparison to existing literature, including consideration of prehabilitation programs (pre-treatment exercise).

• Please discuss whether there are studies with contrasting results (e.g., improvements later in chemotherapy cycles).

• Limitations should be expanded. For example, if all sessions occurred at gyms, patients who were well enough to travel may be overrepresented, introducing bias. Were gym memberships provided? How might access have differed for participants living farther away?

• The implications of the findings remain unclear. What concrete recommendations arise from this study, and how might they influence clinical or community practice?

• The mention of “support” for participants is vague- please clarify what type of support is meant, how it is provided, and by whom.

Reviewer **4**

Name **Symanowski, James**

Affiliation **Levine Cancer Institute, Department of Cancer Biostatistics**

Date **16-Oct-2025**

COI **None**

Dear Authors.

The current research examines adherence to endurance and resistance training across and within chemotherapy cycles in breast cancer patients receiving neoadjuvant or adjuvant chemotherapy.

Comments and recommended changes follow.

1. On line 174, "In Phys-can participants increased their planned exercise volume..." reads confusing. Perhaps this sentence should be "Phys-can participants increased their planned exercise volume...".

2. On line 200, it appears that "A modified Balke protocol" should read "A modified Bjork protocol" based on reference 15. Please clarify.

3. Throughout the manuscript, when reporting results, it is not clear what σ**2 means. this is not previously described. Typically, the standard error of the mean or the standard deviation is reported.

4. Lines 309 through 312 raises a very interesting point. Perhaps receiving supportive therapies should have been included in the modeling. If not, then please clarify.

5. In Table 1, it is appreciated that "missing data" lines for each factor are included. However, it is not clear why there was so much missing data. The current research is utilizing a consent-based clinical trial (the Phys-Can RCT). Please clarify why there was this degree of missing data stemming from this well design Phys-Can RCT.

VERSION 1 - AUTHOR RESPONSE

Reviewer: 1
Dr. Ayako Okuyama, St Luke's International University, National Cancer Center Japan
Comments to the Author:
Adherence to an exercise intervention during (neo-) adjuvant chemotherapy in patients with

breast cancer-data from the Phys-Can RCT

Thank you for giving me an opportunity to review the manuscript. This study deals with the interesting topic of exercise in patients undergoing chemotherapy. However, there are concerns about publishing the results of the RCT.

Major concern

1) By way of background, the authors note that research on exercise adherence in breast cancer patients is limited. However, the GAPs, such as what is known and what would be useful to investigate, are not adequately communicated to the reader.

Authors response: We appreciate your insightful comments and suggestions for revising this manuscript. The introduction section has been updated to highlight research limitation in existing knowledge. We refer to page 4, lines 106-117 for details.

2) It is not clear what is being evaluated as the primary outcome in this study. It says it wants to look at the effects of fatigue, or describes it as exploring factors, which confuses the reader.

Authors response: Thank you for pointing this out. This has been clarified in the abstract and method section p. 5, line 123-132, and p6 line 195 in the revised version.

3) In the methods, it is necessary to state what was assessed as the primary outcome and how the sample size was estimated.

Authors response: The primary outcome has now been clarified in the abstract and in the Methods section (p. 6, line 195). Please note that the Phys-Can trial did not include a sample size estimation for the adherence outcome. This is because adherence to exercise was a secondary outcome in the Phys-Can trial, and the present study does not address the trial's primary outcome of cancer related fatigue, please see Demmelmaier et. al. for results on the main outcome cancer related fatigue.

Demmelmaier I, Brooke HL, Henriksson A, Mazzoni A, Bjørke ACH, Igelström H, et al. Does exercise intensity matter for fatigue during (neo-)adjuvant cancer treatment? The Phys-Can randomised clinical trial. Scandinavian Journal of Medicine & Science in Sports. 2021 Feb;sms.13930.

4) Although the exclusion criteria for selecting subjects have been described, it is necessary to first describe the selection criteria in the first place. Breast cancer may be treated with a variety of chemotherapies. The purpose of chemotherapy (e.g., prevention of recurrence, arrest of progression, etc.) may also vary. It is necessary to clearly state which patients were selected for intervention. Furthermore, older patients may have difficulty exercising in the first place. It also needs to be clearly stated how the patients were selected, taking age and other factors into consideration.

Authors response: Thank you for pointing this out. We have clarified the treatment intent for the included participants on p. 5, lines 140–142. We did not exclude participants based on age, as age does not necessarily reflect physical ability. Instead, exclusion was based on 'inability to perform activities of daily living' and 'disabilities that prevented physical activity.' For clarity, we have added that the treating oncologist or surgeon assessed the inclusion and exclusion criteria (see p. 5, line 149).

5) It is also necessary to explain who guided the intervention and how it was performed by the patient.

Authors response: Thank you for this helpful suggestion. The requested information has been added to the Exercise Intervention section on p. 5, lines 156–158.

6) Please explain the method of recruiting the target patients.

7) Please clarify this objective and then consider the discussion.

Authors response: Participants were consecutively included in the Phys-Can trial. This has been clarified under materials and methods p. 5 line 129.

Minor
Why not add a graph to show sample assignments and dropouts?
Authors response: Thank you for the suggestion. The CONSORT diagram for the Phys-Can trial, including sample assignments and dropouts, has already been published in the main trial publication. As this study is a secondary analysis, we have therefore referred readers to the previously published CONSORT diagram rather than reproducing it here.

Reviewer: 2
Dr. Elizabeth S Dylke, The University of Sydney
Comments to the Author:
There is growing evidence of the benefits of undertaking exercise during treatment for breast cancer and understanding the barriers to completing physical activity are worthy of consideration. The treatment for cancer is physically difficult, potentially leading to challenges in maintaining physical activity throughout chemotherapy treatment.
This manuscript provides some novel information to the field but would benefit from some clarifications to provide better understanding for the reader about the methods and patients as well as something other things to consider in the discussion.

General feedback
Through the article the phrasing used is “(neo-)adjuvant” likely reflecting that some patients had their chemotherapy before surgery and others had it after surgery. However, its actually never explained why that term is used, nor is it described in the participant table how many people had neoadjuvant chemotherapy and how had many adjuvant chemotherapy. This should be clarified and explained. For example, if some participants are starting chemotherapy not long after surgery, this may impact on their baseline fitness and what they are able to do.
Authors response: Thank you for pointing this out. We have clarified the treatment intent for the included participants on p. 5, lines 141–142. Number of individuals receiving neo-adjuvant vs adjuvant was added to Table 1.

Specific feedback
Methods
The manuscript needs a statement about where the ethics for this project was attained from
Authors response: Thank you for your comment. The Phys-Can trial was approved by the Swedish Ethical Review Authority (Registration number: 2014/249). This information is stated in the Ethics Declarations and Trial Registry Information section.

Exercise intervention
I found this section a bit difficult to understand, without visiting the other paper on the study. I think the part that needed a bit more explanation is the impact of the familiarisation program as it impacts on the results later.
Pg 6, line 137-139: Also, a six-week familiarisation period for resistance training and three-weeks for endurance training, preceded the start of the exercise program according to the protocol 11.
- Please confirm that taking part in this familiarisation program would at times mean that the exercise intervention didn’t start until after the patient had undergone a round or maybe 2 of chemotherapy. What happened during the familiarisation sessions? What was the adherence through this period?
Authors response: Thank you for pointing this issue out. We have added information on the what the familiarisation period entails and that exercise adherence (i.e., exercise volume) could not be calculated for this period. See p 6, line 161-163, and p 7 line 206.

Pg 6: Supervised resistance training- can you confirm how long the participants were supervised for?
Authors response: Thank you for seeking clarification. Participants were supervised throughout the entire 6-month intervention period for those receiving adjuvant chemotherapy, and for 4 months for women receiving neo-adjuvant chemotherapy (as the intervention began during chemotherapy). Each supervised resistance training session lasted approximately 60-90 minutes, including warm-up, the six prescribed exercises, and cool-down. This information has been clarified in the Exercise Intervention section of the revised manuscript.

Pg 7: High-intensity intervals at 80-90% HRR is very high for many healthy participants, let alone those going through a major treatment like chemotherapy. What was the justification for this level of intensity? I also think that in the discussion the impact of the high level of intensity on adherence may be worth considering. (I say this with not only experience as an exercise professional but also as a runner who has gone through chemotherapy for breast cancer and was able to maintain running through it).
Authors response: Thank you for this thoughtful comment and for sharing your personal perspective. The Phys-Can trial was designed to evaluate whether high-intensity exercise had a superior effect on cancer-related fatigue compared to low-to-moderate intensity exercise. The use of 80–90% HRR for the high-intensity endurance training intervals was therefore based on the trial's primary hypothesis that higher intensities might yield greater improvements in fatigue. Although this intensity level is demanding, particularly for individuals undergoing chemotherapy, the exercise sessions were closely monitored, and intensity was continuously adjusted according to each participant's capacity. We agree that the high intensity required may have influenced adherence, and we have added a statement in the Discussion and Interpretation to acknowledge the potential impact of the intervention's intensity on endurance training adherence. See p 10, line 345-346.

Line 176-178: If a participant had to temporarily lower their weights due to side effects, this was considered a decrease in adherence.
- I think more explanation for this decision is needed; if they were able to meet the volume/intensity prescribed based on their current abilities (likely decreased to ensure they were safe in doing the exercise) why is this considered a decrease in adherence. Particularly in a population where they are, because of the treatment they are undertaking, being systematically weakened, it feels like only considering progression as adherence is unreflective of realistic goals for patients. I'd be curious about what percentage of "non-adherent" patients this applied to in your sample and if you considered people doing the dosage you prescribed them, even if it did have to be decreased due to treatment side effects, what the difference in your findings would be.
Authors response: Thank you for raising this important point. We appreciate the concern that classifying reduced training loads during chemotherapy as decreased adherence may appear inconsistent with the realities of treatment-related side effects. We have now clarified in the Methods section that decreases in adherence were defined relative to each participant's most recently recorded training loads. Thus, a lack of progression was not considered non-adherence. Instead, adherence was evaluated based on whether participants were able to complete the loads that were individually prescribed for them at that point in the program. Because participants typically increased their training loads as they became stronger, the prescribed "target weights" were progressively adjusted. When side effects required a temporary reduction in load below this individualized target, this was captured as a decrease in adherence, reflecting a deviation from the planned progression rather than a failure to maintain baseline ability. See p 7 line 209-211.

Results
The results are a bit difficult to follow and seem to have varying numbers that are hard to rectify across different sections.

For example:
Line 251-253: the mean adherence for resistance training across chemo was 58% (39) for HI and 62 % (38) for LMI
Line 268- 272: For resistance training (see Table S2, Model 3) the average level of adherence at week 6 was 73% (, 95% CI [68.79, 76.44], $\sigma 2$ = 196.71, 95% CI [109.64, 323.51]). The average adherence decreased 5 % per week (linear slope = -5.20, 95% CI [-6.85, -3.59], $\sigma 2$ = 33.85, 95% CI [17.38, 59.25]) but levelled off across the treatment period (quadratic slope = 0.38, 95% CI [0.22, 0.54], $\sigma 2$ = 0.30, 95% CI [0.13, 0.57]).
- I understand that because of the familiarisation period, week 6 is the starting point for a lot (but I'm not sure how many) of participants (does this correspond to the end of the 2 round of chemo- I don't really understand how the weeks compare across individuals in the study). Starting at 73% adherence and then decreasing at 5% per week across 12 weeks, seems like it should end at a lower overall average adherence of 58%/62%. What do you mean that the decrease in adherence levelled off across the treatment period in reference to the average decrease?
Authors response: Thank you for seeking clarification. The descriptive mean adherence values (58% for HI and 62% for LMI) were simple averages calculated across the entire chemotherapy period and were intended to provide broad contextual information. However, these values do not correspond to any specific study aim and are not directly comparable to the model-derived estimates. Regarding the wording "levelled off," this refers to the significant positive quadratic term in the model, indicating that although adherence initially decreased at approximately 5% per week, the rate of decline slowed over subsequent weeks rather than continuing linearly. This nonlinear pattern results in a trajectory that does not mirror the simpler descriptive average of 58–62%. To avoid confusion and better align with the study aims, we have removed the descriptive adherence values from the revised manuscript and now report only the model-based results (p.9, line 295-298 will be removed in the revised manuscript).

Why was baseline fatigue the factor that was included in the determination of factors that may impact adherence? Was there differences in baseline fatigue between those who had adjuvant v neo-adjuvant chemo? Were there differences in adherence between those who had adjuvant v neo-adjuvant chemo?
Authors response: Thank you for your comment. Fatigue was included because it is one of the most common treatment-related side effects and has previously been shown to influence exercise adherence. Baseline comparisons between the exercise-intensity groups have been conducted to ensure that potential confounding factors, including fatigue, were balanced between groups (see revised Table 1). Only approximately 13% of participants received neoadjuvant chemotherapy, and we therefore did not conduct subgroup analyses comparing baseline fatigue or adherence between the adjuvant and neoadjuvant groups.

Discussion
General comments- I think your adherence rates are pretty impressive. It might be good to also mention what adherence rates for this type of program (or physical activity programs) is for healthy people of a similar age. I think this discussion should actually be scaffolded in more positive sense. More details on how much supervision/support participants got during the trial would help others know how implementable this approach would be.
Authors response: We appreciate the reviewer's positive assessment of the adherence rates. However, we believe that comparing our adherence rates to those in other cancer populations undergoing chemotherapy is most clinically relevant. Studies in breast cancer patients show similar declining patterns during chemotherapy treatment (refs 5, 8, 9), and adherence challenges are likely driven by treatment-specific factors (e.g., cumulative fatigue, neutropenia, nausea) that do not affect healthy populations. Comparisons to healthy individuals would therefore be less informative for understanding adherence barriers during cancer treatment or for developing targeted clinical

interventions. We have expanded our Discussion to better contextualise our findings within the existing cancer exercise literature.

Also as mentioned earlier, commenting on the impact of the type of program (particularly for the HI endurance program, which appeared to have a greater decrease in adherence across the time period) would be worthwhile. This is a very tough program that you've asked participants to do.

Line 319-321: Increasing fatigue levels within chemotherapy cycles, or fatigue accumulated over the treatment period, may play a more important role in adherence than fatigue at the start of treatment
- As this study was designed to investigate fatigue, can you look into this with your data?
Authors response: We appreciate this suggestion. While the Phys-Can trial included detailed fatigue assessments, these were conducted at baseline, 3 months, and 6 months rather than weekly throughout chemotherapy cycles. Therefore, we do not have the temporal resolution needed to examine how fatigue fluctuates within individual 3-week chemotherapy cycles or to relate these fluctuations to weekly adherence patterns. This represents an important area for future research, and we have noted this as a research priority in the revised Discussion, p 11 line 377.

Other limitations- other chemotherapies are also given in 3 weekly cycles but may be associated with other/more barriers to maintaining exercise levels due to side effects associated with them
Authors response: Thank you for point this out, we clarified this under strength and limitations. p 12, line 410-411.

Reviewer: 3
Dr. Gillian Gresham, Cedars-Sinai Medical Center Samuel Oschin Comprehensive Cancer Institute
Comments to the Author:
Thank you for the opportunity to review this manuscript. The topic is interesting and relevant. However, major revisions are needed to improve the clarity of the manuscript, the quality of the writing, and the overall reporting of methods and results. My detailed comments are outlined below:

Title
• The title could be refined to better reflect the study aims and findings.
Abstract
• Methods: Consider presenting the study design, data source, and study population first, followed by details of the statistical analysis. More information should also be provided about the intervention, control group, and outcomes assessed.
• Results: Please clarify whether results reflect differences between study arms or are reported across the entire sample.
Strengths and Limitations
• The current statement, "Novel and detailed adherence reporting providing detailed insights," is vague. Please clarify what novel insights are provided and how they add to existing literature.
Authors response: Thank you for your comments. We have revised the title of the manuscript and the abstract according to Instruction for authors and the Consort extensions for abstracts. See Title page and p. 2 line 68-73. Also, we have removed the novelty statement in the strength and limitations sections because it is not supposed to be included in the strength and limitations.

Manuscript
Introduction
• The writing throughout could be improved to enhance clarity and flow, and typographical errors should be corrected.

Authors response: Thank you for pointing this out. The manuscript has been thoroughly proofread again by a native English speaking co-author to improve clarity, flow, and correct typographical errors.

• The aims should be framed within the broader context of the Phys-Can multicenter trial, which should be introduced earlier in the introduction with appropriate references.
Authors response: Thank you for pointing this issue out. We have now clarified this in the introduction section, p. 4 line 106. In addition, more information about the Phys-Can trial have been added in the Materials and methods section.

Methods
• It is unclear whether this is a secondary analysis of the Phys-Can trial or a prospective sub-study built into the trial. Please clarify this distinction, including whether additional consent was obtained for this subset of participants.
Authors response: This is a secondary analysis (i.e., adherence was not the main aim of Phys-Can). This information has now been clarified in the abstract and in the Methods section (p. 6, line 179).

• Eligibility criteria should be clarified: were they the same as the parent trial or different?
Authors response: The adherence data from the women with breast cancer participated in the Phys-Can RCT were analysed in this study. This information has now been clarified in the abstract and in the Materials and methods section (p. 5, line 131-133).

• Intervention details need further elaboration:
o The manuscript states the intervention was supervised at a gym, but by whom?
Authors response: Thank you for highlighting the need for additional detail. The requested information regarding who supervised the gym-based sessions has now been added to the Exercise intervention section on p. 6, lines 158–159

o How was adherence monitored and tracked?
Authors response: Adherence data were based on logbook data for resistance training and a combination of logbook data and objective heart rate data for endurance training. This information is added to the manuscript, p 6, line 170-173 and 183-185.

o How were individual exercise intensity levels assessed and adjusted?
Authors response: Information with regard to this was added to the manuscript, see p 6, line 170-173 and 183-185.
·

• Outcome variables: Please clarify how adherence was assessed (e.g., self-report, physiologist evaluation) and how it was analyzed. Were any objective measures of physical activity or adherence assessed?
Authors response: Adherence data were based on logbook data for resistance training and a combination of logbook data and pulse file data for endurance training. This information is added to the manuscript, p 6, line 170-173 and 183-185.

• Predictor variables: Consider whether chemotherapy timing within cycles was evaluated.
Authors response: We appreciate the opportunity to clarify our analytical approach. For the within-cycle analysis, we collapsed data across all six chemotherapy cycles to examine average adherence patterns during week 1, week 2, and week 3 of each 3-week cycle. For example, adherence data from the first week of cycle 1, the first week of cycle 2, through the first week of cycle 6 were averaged to represent "cycle week 1." This approach allowed us to examine whether adherence systematically varied within the recurring 3-week treatment structure while accounting for the overall declining trend across the full 18-week period (through detrending).

Performance status (e.g., KPS or ECOG), treatment side effects/toxicity, and baseline PROs or quality of life measures could also be important predictors and should be addressed.
Authors response: We selected predictor variables based on the data available within the Phys-Can trial and informed by previous research in this area. Although formal performance status measures were not evaluated, we believe that most participants had a high-performance status, as all were eligible for neoadjuvant or adjuvant chemotherapy and none met the exclusion criteria related to functional limitations.

Results
• Results should be described in greater detail overall.
• A CONSORT-style flow diagram would strengthen the manuscript by showing the number of participants from the Phys-Can trial, those included in the present analysis, and reasons for exclusion (especially if different from the parent trial)
Authors response: Thank you for the suggestion. The CONSORT diagram for the Phys-Can trial, including sample assignments and dropouts, has already been published in the main trial publication. As this study is a secondary analysis, we have therefore referred readers to the previously published CONSORT diagram rather than reproducing it here.

• Baseline characteristics should be summarized in the text (Table 1) and compared across exercise groups.
Authors response: We have added a baseline comparison between exercise intensity groups in table 1 and information on the results in the beginning of the Results section, p.9, lines 292-295.

• More detail is needed regarding baseline predictors (e.g., whether baseline fatigue was associated with outcomes).
Authors response: As seen in table 2, 3 and 4 baseline fatigue were not associated with adherence across the treatment period for neither resistance or endurance training.

• Were longitudinal changes in predictors such as fatigue or muscle strength assessed across chemotherapy cycles, and were these associated with adherence outcomes?
Authors response: Unfortunately, we did not have access to data on fatigue across chemotherapy cycles, although we agree that this would have been valuable to assess. We measured 1RM only at baseline and after the exercise intervention. However, 6RM and 10RM were assessed for all strength-training exercises every 4–6 weeks to adjust training loads, and these measures are likely associated with adherence.

• Sentence at lines 331–332 is unclear and incomplete and should be revised.
Authors response: Thank you for your comment. We have now revised the sentence to: "However, we observed that higher baseline cardiorespiratory fitness was associated with higher resistance training adherence during the first week (week 1) of the chemotherapy cycle." See p. 11 lines 361-363.

Discussion/Conclusion
• The discussion would benefit from deeper comparison to existing literature, including consideration of prehabilitation programs (pre-treatment exercise).
Author response: We have expanded the Discussion to include a deeper comparison with existing literature, including consideration of prehabilitation programs. Specifically, we now discuss how pre-treatment exercise may improve cardiorespiratory fitness and potentially support exercise capacity during chemotherapy, drawing on findings from a systematic review.

• Please discuss whether there are studies with contrasting results (e.g., improvements later in chemotherapy cycles).
Author response: To our knowledge, this is the first study to examine exercise adherence among women with breast cancer across the entire chemotherapy treatment period and within individual chemotherapy cycles. Previous studies that have assessed adherence at multiple time points, rather than reporting only mean adherence across treatment, have shown similar patterns, with adherence generally declining as chemotherapy progresses. At present, we are not aware of any published studies reporting contrasting findings, such as improvements in adherence later in chemotherapy.

• Limitations should be expanded. For example, if all sessions occurred at gyms, patients who were well enough to travel may be overrepresented, introducing bias. Were gym memberships provided? How might access have differed for participants living farther away?
Author response: All resistance training sessions in the Phys-Can trial were conducted at gyms, and participants needed to live close enough to travel to the gym. Gym memberships were provided to participants, but for those living fart from the training sites, travel distances may have affected adherence. We have expanded the Limitations section in the manuscript to acknowledge that these factors may influence generalizability. This was added at p 11 line 388-392.

• The implications of the findings remain unclear. What concrete recommendations arise from this study, and how might they influence clinical or community practice?
• The mention of "support" for participants is vague- please clarify what type of support is meant, how it is provided, and by whom.
Author response: In the Interpretation section, p 12, lines 424-426, we have specified the implications, including the definition of support.

Reviewer: 4
Dr. James Symanowski, Levine Cancer Institute
Comments to the Author:
Dear Authors.
The current research examines adherence to endurance and resistance training across and within chemotherapy cycles in breast cancer patients receiving neoadjuvant or adjuvant chemotherapy.

Comments and recommended changes follow.

1. On line 174, "In Phys-can participants increased their planned exercise volume..." reads confusing. Perhaps this sentence should be "Phys-can participants increased their planned exercise volume...".
Authors response: Thank you for pointing this out. We agree that the phrasing was unclear. The sentence has now been revised to: "Phys-Can participants increased their planned exercise volume" as suggested to improve readability.

2. On line 200, it appears that "A modified Balke protocol" should read "A modified Bjork protocol" based on reference 15. Please clarify.
Authors response: Thank you for noting this. The correct term is indeed the *modified Balke protocol*, and reference 15 describes this protocol. We have reviewed the citation and clarified the wording in the manuscript to avoid confusion see p.7, line 233-234.

3. Throughout the manuscript, when reporting results, it is not clear what σ**2 means. this is not previously described. Typically, the standard error of the mean or the standard deviation is reported.

Authors response: In the manuscript, σ^2 denotes the variance, which is the square of the standard deviation and describes the variability of the data around the mean. To improve clarity, we have added additional explanation of this notation and its interpretation in the Statistical Analysis section p 8, line 262-265.

4. Lines 309 through 312 raises a very interesting point. Perhaps receiving supportive therapies should have been included in the modeling. If not, then please clarify.
Author response: We agree that receiving colony-stimulating growth factors could potentially influence exercise adherence through reduced neutropenia, and might be relevant to include in the modelling. In our sample all individuals receiving high-dose chemotherapy also received colony-stimulating growth factors why these variables would have correlated if added to the models.

5. In Table 1, it is appreciated that "missing data" lines for each factor are included. However, it is not clear why there was so much missing data. The current research is utilizing a consent-based clinical trial (the Phys-Can RCT). Please clarify why there was this degree of missing data stemming from this well design Phys-Can RCT.
Author response: Some missing data occurred for variables included in this secondary analysis that were not part of the primary Phys-Can outcome (which was cancer-related fatigue). Additionally, ongoing chemotherapy occasionally prevented participants from completing certain physical assessments (e.g., 1RM, VO_2max, BMI) due to treatment-related side effects or scheduling difficulties.

VERSION 2 - REVIEW

**Reviewer**	**2**
**Name**	**Dylke, Elizabeth S**
**Affiliation**	**The University of Sydney**
**Date**	**06-Jan-2026**
**COI**	

Thank you to the authors for the changes they have made; they have strengthened the article. There remains a few changes and things to consider in the interpretation of your findings.

Abstract

Line 53: CIs is short for confidence interval not credible intervals

Results

Specific comments

Pg 9, line 294-295: “About 13% of participants received neo-adjuvant chemotherapy.”

- Consider putting specific numbers of each group instead of “about”

Figure 1a & b

Is this the average level of adherence? If so, should be in the figure title. The values also appear to be different from the text- e.g. adherence for resistance training appears to be closer to 80% when the text says 73%. If the figure isn't average adherence than this might explain the difference.

Figure 2

I don't really understand what data this graph is presenting!

Is this average decline in adherence or is average adherence between weeks? The label on the y axis doesn't appear to link with the figure title. The CI presented in the results section appear to be quite large but the error bars here are very tight.

Discussion

General comment: In reading this article again, I think another important consideration is that with the adherence that the participants had, the group had no significant decrease in strength or fitness. The level of physical activity they were able to maintain through the ups and downs of chemo was able to maintain their baseline levels. If the goal is maintenance of fitness, this evidence might suggest that a lower level of physical activity is sufficient. Do we need to push patients to try reach the higher level of weekly activity? This is also supported by the fact that the lower intensity group had better adherence.

Specific comments:

Pg 11, line 386-388: "Also, exercise may offer patients short term improvements of energy levels and nausea, why informing and encouraging patients to engage in exercise despite side-effects may be helpful."

- Sentence is missing something

**Reviewer**	**4**
**Name**	**Symanowski, James**
**Affiliation**	**Levine Cancer Institute, Department of Cancer Biostatistics**
**Date**	**28-Dec-2025**
**COI**	

Dear Author.

Thank you for submitting your revised manuscript. The responses to my initial review comments and to those from the other reviewers were informative. The associated updates to the revised manuscript (when applicable) have improved the overall strength and readability of this manuscript.

VERSION 2 - AUTHOR RESPONSE

Reviewer: 2
Dr. Elizabeth S Dylke, The University of Sydney
Comments to the Author:

Thank you to the authors for the changes they have made; they have strengthened the article. There remains a few changes and things to consider in the interpretation of your findings.

Abstract
Line 53: CIs is short for confidence interval not credible intervals

Thank you for your careful reading of our manuscript and for highlighting this point. You are correct that "CI" is commonly used as an abbreviation for confidence intervals. However, because our analysis is based on Bayesian multilevel growth curve models, the appropriate term is *credible intervals*. To avoid confusion, we have revised the abbreviation to "CrI" throughout the manuscript.

Results
Specific comments
Pg 9, line 294-295: "About 13% of participants received neo-adjuvant chemotherapy."
- Consider putting specific numbers of each group instead of "about"

Thank you for pointing this out. The sentence now reads: ""12.9% of participants received neo-adjuvant chemotherapy (n=23).""

Figure 1a & b
Is this the average level of adherence? If so, should be in the figure title. The values also appear to be different from the text- e.g. adherence for resistance training appears to be closer to 80% when the text says 73%. If the figure isn't average adherence than this might explain the difference.

Authors response: Thank you for this observation. Figure 1 presents the observed mean adherence at each week, whereas the value reported in the text (73%) refers to the intercept from the Bayesian multilevel growth model (i.e. the model-estimated adherence at the reference time point). Because the model uses the full longitudinal trajectory and includes all participants, including those with missing observations, this estimate is not expected to exactly match the observed weekly means shown in the figure. The weekly means are presented to provide a descriptive visualisation of adherence patterns across the 18-week treatment period. We have now clarified this in the Figure 1 caption.

Figure 2
I don't really understand what data this graph is presenting!
Is this average decline in adherence or is average adherence between weeks? The label on the y axis doesn't appear to link with the figure title. The CI presented in the results section appear to be quite large but the error bars here are very tight.

Author response: Thank you for this comment. Figure 2 presents the model-estimated marginal mean adherence (%) at Weeks 1–3 within the chemotherapy treatment cycle, from which the pattern of decline across weeks can be observed, rather than a direct estimate of average decline itself. We agree that the previous title was potentially misleading and have revised the figure title and caption to clarify this. The error bars represent credible intervals around the model-estimated means, whereas the credible intervals reported in the Results section relate to model parameter estimates and are therefore not directly comparable.

Discussion
General comment: In reading this article again, I think another important consideration is that with the adherence that the participants had, the group had no significant decrease in strength or fitness. The level of physical activity they were able to maintain through the ups and downs of chemo was able to maintain their baseline levels. If the goal is maintenance of fitness, this evidence might suggest that a lower level of physical activity is sufficient. Do we need to push patients to try reach the higher level of weekly activity? This is also supported by the fact that the lower intensity group had better adherence.

Author response: Thank you for this thoughtful comment. We agree that this is an important and clinically relevant consideration. However, our study was not designed to determine the minimal effective dose of exercise required to maintain or improve physical fitness during chemotherapy. Therefore, interpreting the observed adherence (i.e. adherence below 100%) as evidence that a lower exercise volume is sufficient to maintain or improve outcomes such as physical fitness would extend beyond what our data can directly support. Addressing this question would require studies specifically designed to examine dose–response relationships and the minimal effective exercise dose during chemotherapy.

Specific comments:
Pg 11, line 386-388: "Also, exercise may offer patients short term improvements of energy levels and nausea, why informing and encouraging patients to engage in exercise despite side-effects may be helpful."

- Sentence is missing something

Author response:
Thank you for pointing this out. We agree that the original sentence was incomplete and unclear, and we have now revised it for clarity. The revised sentence reads (page 11, lines 384–386): *"Exercise may offer patients short-term improvements in energy levels and reductions in nausea. Consequently, healthcare providers should inform and encourage patients to engage in physical activity, even when side effects are present."*
